# Antimalarial Quinoline Drugs Inhibit β-Hematin and Increase Free Hemin Catalyzing Peroxidative Reactions and Inhibition of Cysteine Proteases

**DOI:** 10.1038/s41598-019-51604-z

**Published:** 2019-10-28

**Authors:** Tomás Herraiz, Hugo Guillén, Diana González-Peña, Vicente J. Arán

**Affiliations:** 1 Instituto de Ciencia y Tecnología de Alimentos y Nutrición (ICTAN). Spanish National Research Council (CSIC), Juan de la Cierva 3, 28006, Madrid, Spain; 2 Instituto de Química Médica (IQM-CSIC), Juan de la Cierva 3, 28006 Madrid, Spain

**Keywords:** Mechanism of action, Proteases, Drug screening

## Abstract

Malaria caused by *Plasmodium* affects millions people worldwide. *Plasmodium* consumes hemoglobin during its intraerythrocytic stage leaving toxic heme. Parasite detoxifies free heme through formation of hemozoin (β-hematin) pigment. Proteolysis of hemoglobin and formation of hemozoin are two main targets for antimalarial drugs. Quinoline antimarial drugs and analogs (β-carbolines or nitroindazoles) were studied as inhibitors of β-hematin formation. The most potent inhibitors were quinacrine, chloroquine, and amodiaquine followed by quinidine, mefloquine and quinine whereas 8-hydroxyquinoline and β-carbolines had no effect. Compounds that inhibited β-hematin increased free hemin that promoted peroxidative reactions as determined with TMB and ABTS substrates. Hemin-catalyzed peroxidative reactions were potentiated in presence of proteins (i.e. globin or BSA) while antioxidants and peroxidase inhibitors decreased peroxidation. Free hemin increased by chloroquine action promoted oxidative reactions resulting in inhibition of proteolysis by three cysteine proteases: papain, ficin and cathepsin B. Glutathione reversed inhibition of proteolysis. These results show that active quinolines inhibit hemozoin and increase free hemin which in presence of H_2_O_2_ that abounds in parasite digestive vacuole catalyzes peroxidative reactions and inhibition of cysteine proteases. This work suggests a link between the action of quinoline drugs with biochemical processes of peroxidation and inhibition of proteolysis.

## Introduction

Malaria is a parasitic infection caused by *Plasmodium* that affects hundreds millions people worldwide and causes almost half a million deaths each year^1^. It remains a major infectious disease due to the lack of an effective vaccine and widespread resistance to available drugs. During infection, *Plasmodium* passes over several stages including an intraerythrocytic stage, in which parasite degradates 60–80% of host hemoglobin that is used as food support for its development and growth. Hemoglobin is oxidized to methemoglobin within parasite digestive vacuole and is hydrolyzed by aspartic proteases into free heme (Fe^3+^) (ferriprotoporphyrin IX) and denatured globin. Globin is hydrolyzed by cysteine proteases (*i.e*. falcipains) and exopeptidases into small peptides and amino acids used for protein synthesis^2–5^. Digestion of hemoglobin releases large quantities of heme (Fe^3+^) that accumulates and reaches high concentrations (up to 300–500 mM). Those high concentrations are thought to be toxic for *Plasmodium* through membrane disruption, lipid peroxidation, and protein and DNA oxidation^2,6–11^. Free heme (Fe^3+^) might also interfere with hemoglobin degradation pathway^12,13^. *Plasmodium* uses a system to detoxify heme (Fe^3+^) called biocrystallization based on the formation of hemozoin pigment which appears as a dark black crystalline spot (a dark brown pigment) in red blood cells of infected patients^14–18^. Hemozoin is chemically and structurally identical to β-hematin, a heme dimer that crystallizes under the acidic conditions of digestive vacuole of *Plasmodium* (pH values of 4.8–5.0)^18–20^. It contains two heme (Fe^3+^) monomers reciprocally linked through coordination complexes between the carboxyl group of a propionate side chain of one monomer and the iron (Fe^3+^) atom in the porphyrin ring of another monomer^19,21^. β-Hematin is stored in crystalline form in the digestive vacuole where it is apparently nontoxic for *Plasmodium*^22^. The process of detoxification of heme is not unique of *Plasmodium* and occurs in other organisms that use hemoglobin such as *Schistosoma mansoni*, and *Rhodnius prolixus*^23^. The mechanisms underlying the formation of β-hematin from hemoglobin are still poorly understood despite this process is vital for the survival of parasite^24^. It appears to be catalyzed by lipids and/or heme detoxification proteins^24–29^ but it also occurs in absence of biological materials^30^.

**Figure 1:**
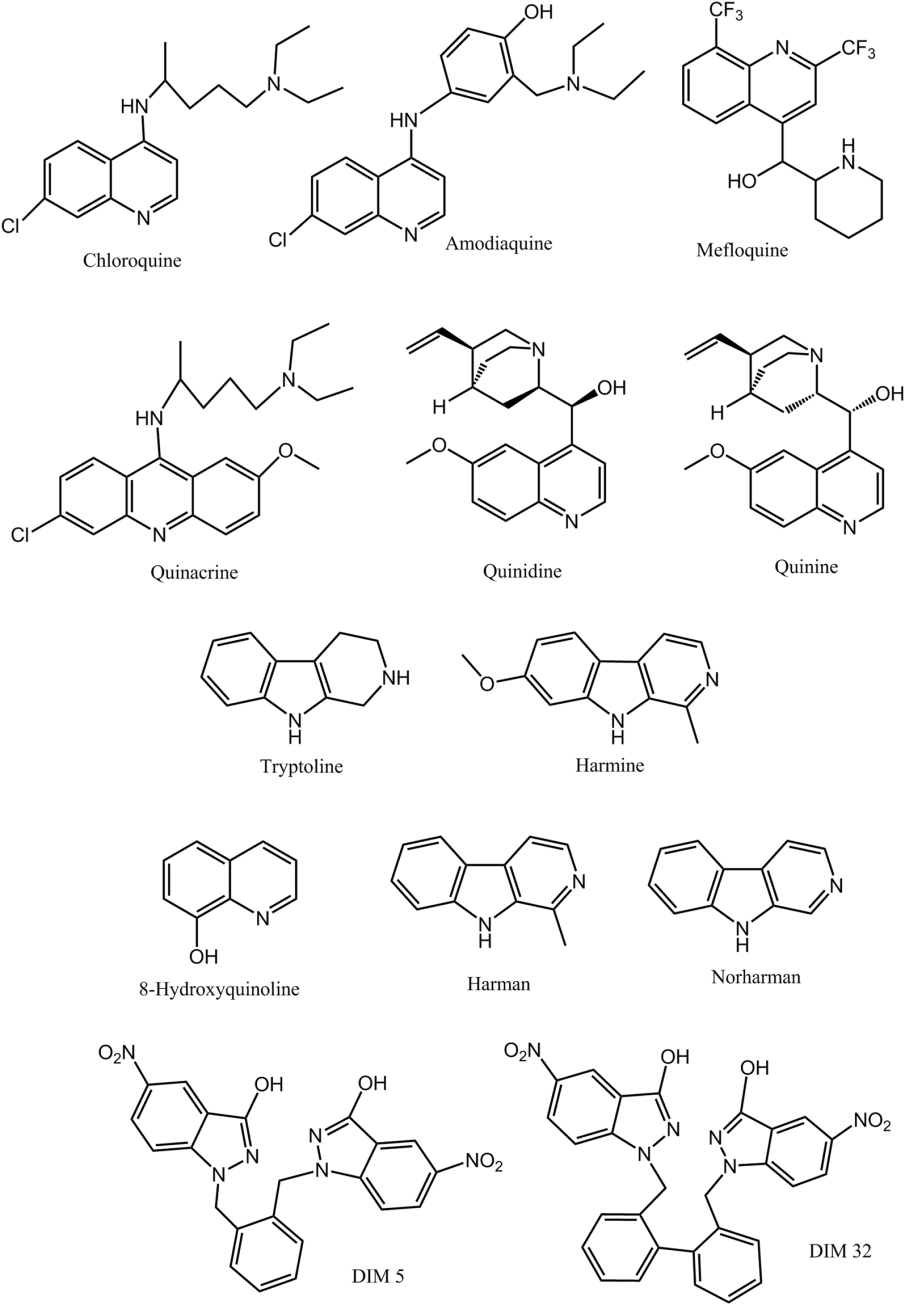
Quinoline drugs, β-carboline alkaloids, and nitroindazole compounds studied as inhibitors of hemozoin (β-hematin).

The synthesis of hemozoin constitutes a unique system of *Plasmodium* to detoxify heme; therefore its inhibition is a useful target for antimalarial drugs action^2,18,31,32^. Quinoline drugs (*e.g*. chloroquine) (Fig. 1) have been successfully used for years to treat malaria. Currently, these drugs are less effective as parasites are becoming resistant. Quinoline drugs exert their antimalarial action by interfering with heme detoxification^33–35^. Nevertheless, their ultimate mechanism of action remains unknown and more insights are needed to understand how these drugs may produce toxic effects to parasite in order to increase their pharmacological efficacy and lower resistances^2,36–41^. Previous studies suggested that quinolines could inhibit peroxidative degradation of heme contributing to build-up of toxic membrane-associated heme that would destroy membranes^42^ whereas others have reported a minor role of quinolines in the degradation of heme^43^. The current work determines the actions of quinoline drugs and related compounds (e.g. β-carbolines and nitroindazoles) having antimalarial activity^44,45^ on the synthesis of hemozoin and free hemin build-up. We have developed an assay to determinate the conversion of hemin into hemozoin, and subsequently it is used to investigate the activity of quinoline drugs. Results show that active quinolines (e.g. chloroquine) increase free hemin that is involved in peroxidative reactions and inhibition of proteolysis. Then, this work highlights a link between free hemin build-up by quinoline drugs with peroxidative effects (oxidative effects) and inhibition of cysteine proteases (inhibition of proteolysis). These events may underlay the basics for toxicity of antimalarial quinoline drugs in blood feeding organisms including *Plasmodium* and could be useful for the development of new antimalarial agents.

## Results

### Formation of β-hematin and inhibition by quinoline drugs

**Figure 2:**
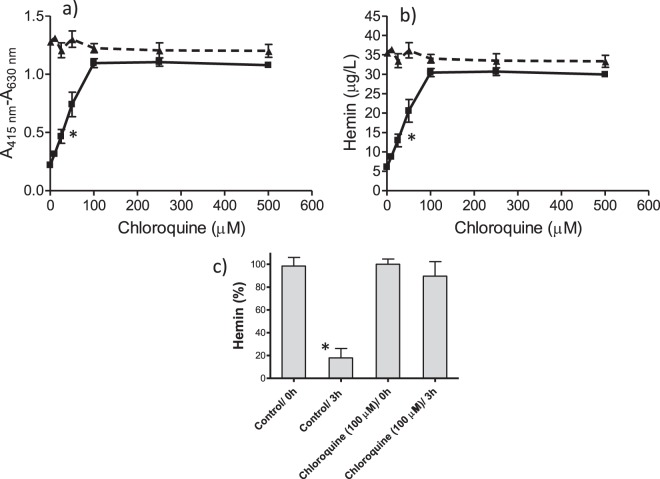
Increase of free hemin (inhibition of β-hematin) in presence of chloroquine. Absorbance (A_415 nm_ − A_630 nm_) (**a**) and concentration of free hemin (**b**) (initial ▲, and after incubation 3 h, ■); and hemin (%) in control and chloroquine (100 µM) (0 and 3 h) (**c**). Hemin was incubated with chloroquine (0–500 µM) at pH 4.8 in presence of tween20. Data are average from seventeen assays. (*) Data are significantly different (p < 0.01) from control without chloroquine for this one and higher concentrations of chloroquine (**a**,**b**), and from control (0 h) (**c**).

**Figure 3:**
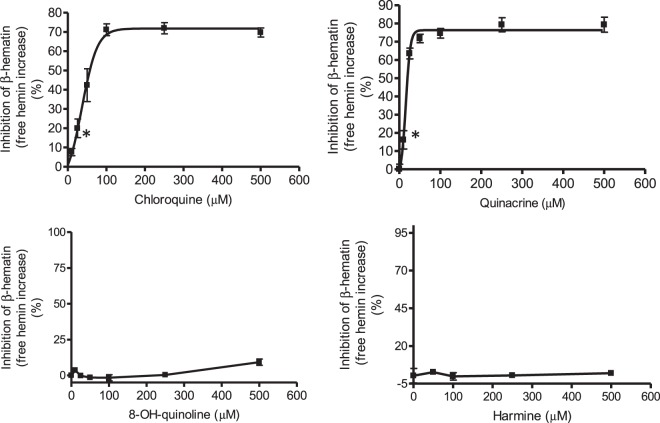
Inhibition (%) of β-hematin (or increase of free hemin) by chloroquine, quinacrine, 8-OH-quinoline and harmine. Data are average ± SEM from seventeen assays for chloroquine, eight for quinacrine, and three for 8-OH-quinoline and harmine, respectively. Basal values in absence of drug are subtracted, and chloroquine and quinacrine are adjusted to non-linear regression curves. (*) Data are significantly different from control without drug for this one and higher concentrations of drug (p < 0.01).

Hemin incubated at 37 °C and pH 4.8 (pH of digestive vacuole) in presence of tween 20 crystallized and precipitated as a dark (black) powder that was isolated and had IR spectra exhibiting bands at 1210, 1663 and 1712 cm^−1^ (Supplementary Figure 1) corresponding to β-hematin or hemozoin, the pigment of *Plasmodium*^18,24,46^. β-Hematin has UV-VIS spectrum with lower relative absorbance at 415 nm and higher at 630 nm than free hemin, so that the concentration and relative contribution of free hemin can be assessed from A_415 nm_-A_630 nm_^47,48^. A high proportion of initial hemin (>80%) is converted to β-hematin during incubation time (Fig. 2). Drugs that inhibit β-hematin proportionally increase free hemin measured as A_415 nm_-A_630 nm_ (Fig. 2a,b). Thus, chloroquine inhibited β-hematin and increased hemin that reached more than 80% of the initial hemin at 100 µM and higher concentrations of drug (Fig. 2b,c). This effect can be seen in photographic image (Supplementary Fig. 2). In presence of chloroquine the hemin remained in solution for hours but it was removed and converted into β-hematin in absence of drug (Supplementary Fig. 3a). The ability of quinolines to inhibit β-hematin, and consequently to increase hemin was subsequently studied (Fig. 3). Table 1 lists the concentration of drug needed to reach a 50% inhibition of β-hematin (i.e. allowing a 50% increase of free hemin) (IC_50_). The most active compounds were quinacrine, chloroquine and amodiaquine whereas quinidine, mefloquine, and quinine had lower activity. The highest inhibition was also produced by quinacrine and chloroquine. 8-Hydroxyquinoline and β-carbolines (norharman, harman, harmine and tryptoline) did not inhibit β-hematin, and consequently did not increase hemin (Fig. 3 and Table 1). Indazole compounds (DIM 5 and DIM 32) gave IC_50_ values comparable to chloroquine in agreement with former results^45^. However, these drugs behaved differently to quinolines as they precipitated hemin which redissolved during assay of absorbance contributing to hemin.

**Table 1:** Inhibition of β-hematin (hemozoin) (i.e. free hemin increase) by quinoline drugs, β-carbolines and nitroindazoles and oxidation of TMB.

Compound	IC_50_ (µM)	Max. inhibition (max. free hemin) (%)^a^	EC_50_ (µM)^b^	Max. oxidation of TMB (%)^c^
Chloroquine	56.9	89.0 ± 3.1	28.1	96.9 ± 3.5
Quinacrine	20.7	96.1 ± 4.1	7.7	94.7 ± 8.5
Amodiaquine	50.1	83.3 ± 2.3	36.6	59.8 ± 3.6
Mefloquine	201	70.2 ± 5.1	40.3	68.9 ± 9.3
Quinine	211	65.9 ± 18	40.2	63.1 ± 7.2
Quinidine	159	82.3 ± 12	47.4	63.8 ± 3.6
8-Hydroxyquinoline	—	21.4 ± 3.7	—	—
Harmine	—	24.5 ± 2.1	—	28
Harman	—	13.0 ± 5.5	—	35
Norharman	—	53.8 ± 9.3	—	25
Tryptoline	—	18.4 ± 4.8	—	26
Nitroindazole DIM 5	17.4	70.7 ± 2.3	—	36.6
Nitroindazole DIM 32	17.0	73.4 ± 1	—	23

^a^Maximum (%) of inhibition of β-hematin or maximum (%) of free hemin reached with drug. Controls without drugs contained a 17.86 ± 1.98% of free hemin remaining from the initial hemin (100%) and the rest converted into β-hematin. ^b^EC_50_ is the concentration of drug allowing to reach a 50% oxidation of TMB. ^c^Maximum oxidation (%) achieved with drug taking the basal oxidation in presence of drug without incubation as 100%. The compounds were tested in a range 0–500 µM except for indazoles that was 0–100 µM.

### Quinolines that inhibit β-hematin increase peroxidation due to hemin build-up

**Figure 4:**
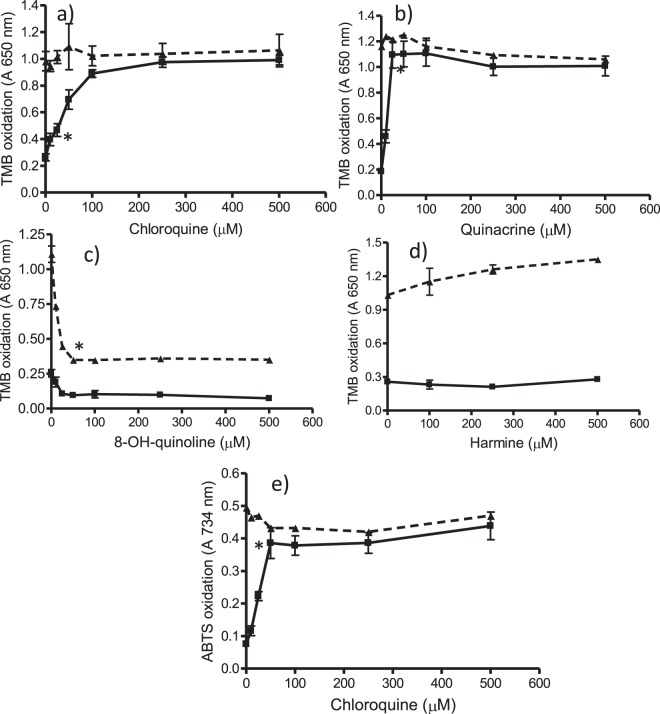
Oxidation of TMB to TMB^+●^ (absorbance at 650 nm) after incubation of hemin with chloroquine, quinacrine, 8-OH-quinoline and harmine for 3 h (■) (**a–d**), and oxidation of ABTS to radical ABTS^●+^ (absorbance at 734 nm) after incubation of hemin with chloroquine for 3 h (■ (**e**). Oxidation in absence of incubation time (t = 0) (-▲-). Oxidation assays were carried out in presence of H_2_O_2_ (1 mM) as indicated in experimental and data averaged seventeen assays for chloroquine, eight for quinacrine, and three for 8-OH-quinoline and harmine. Oxidation of TMB was determined by absorbance at 650 nm (charge-transfer complex or cation radical, TMB^+●^) but similar trend was obtained at 450 nm followed acidification (TMB diimine). (*) Data are significantly different (p < 0.01) from control without drug for this one and higher concentrations of drug.

**Figure 5:**
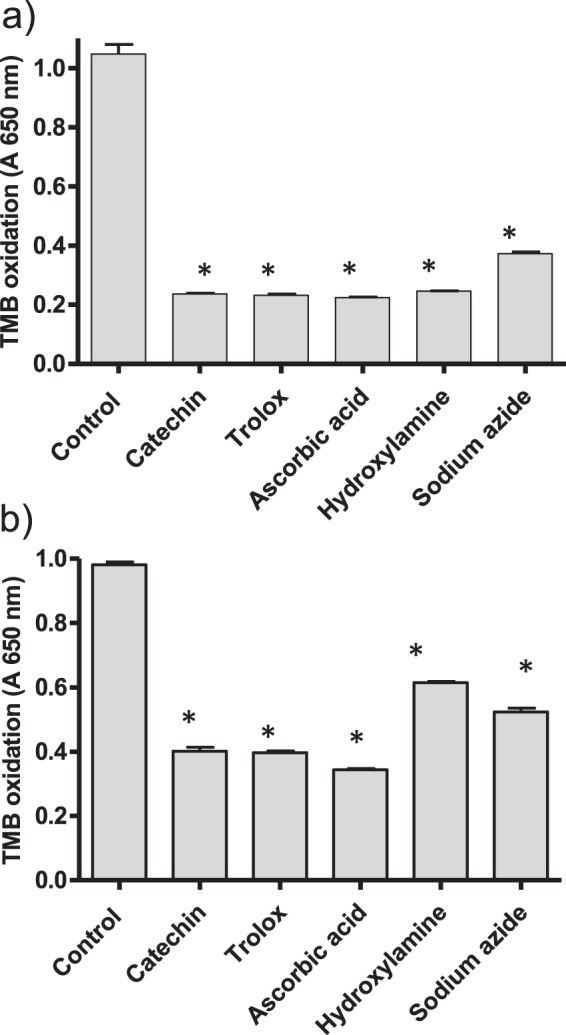
Inhibition of the oxidation of TMB by antioxidants and peroxidase inhibitors. Oxidation of TMB was determined in incubation media (3 h) containing hemin plus chloroquine (100 µM) in presence of H_2_O_2_ (1 mM) and the corresponding antioxidant (1 mM) (**a**); or H_2_O_2_ (100 µM), globin (10 µM) and antioxidant (1 mM) (**b**). Controls had no antioxidant added. Oxidation of TMB to TMB^+●^ cation radical was measured at 650 nm following incubation at 37 °C, 20 min. (*) Data are significantly different from control (p < 0.01).

As shown above, quinolines inhibited β-hematin and increased free hemin. It was studied whether this hemin was able to catalyze peroxidative reactions. For this purpose two peroxidase substrates were used (TMB and ABTS). Active quinolines (*e.g*. chloroquine and quinacrine) increased oxidation of TMB in contrast to inactive compounds (Fig. 4). Moreover, in presence of chloroquine the oxidation of TMB remained high during hours but in absence of drug it decreased simultaneously with hemin (Supplementary Fig. 3b). Peroxidation in presence of those active quinolines was due to free hemin build-up by the action of drugs (Fig. 3 and Fig. 4). Indeed, solutions of hemin standard catalyzed the oxidation of TMB (measured at 650 nm or at 450 nm after acidification) or ABTS (734 nm) in presence of H_2_O_2_ under pH conditions of digestive vacuole (pH 4.8), and oxidation increased with hemin and H_2_O_2_ concentrations (Supplementary Fig. 4). The oxidation in presence of drugs was calculated and EC_50_ values were determined as the amount of drug needed to reach a 50% of initial oxidation (Table 1). The most active compounds were quinacrine (EC_50_ of 7.7 µM) and chloroquine (EC_50_ of 28.1 µM) which also produced the highest levels of oxidation (96.9 and 94.7%, respectively). Quinidine, quinine, amodiaquine and mefloquine gave levels of oxidation lower than 70%. β-Carbolines (norharman, harman, harmine and tryptoline) and nitroindazoles did not increase oxidation whereas 8-hydroxyquinoline decreased basal oxidation owing to antioxidant effects (Fig. 4c). Peroxidation produced by hemin increase in presence of chloroquine was qualitatively similar with ABTS (Fig. 4e) where an EC_50_ of 32.6 µM was measured. Therefore, inhibition of crystallization of hemin (β-hematin) resulted in higher levels of free soluble hemin that catalyzed oxidation of peroxidase substrates in presence of H_2_O_2_. A significant positive correlation was found among free hemin increase (A_415 nm_-A_630 nm_) (Fig. 3) and oxidation of TMB (Fig. 4) for chloroquine (0–500 µM) (correlation coefficient r = 0.99, p < 0.001), quinacrine (0–500 µM) (r = 0.96, p < 0.001), amodiaquine (0–500 µM) (r = 0.98, p < 0.001), mefloquine (0–500 µM) (r = 0.81, p < 0.05), quinine (0–500 µM) (r = 0.84, p < 0.05) and quinidine (0–250 µM) (r = 0.87, p < 0.05). A significant positive correlation was also found among free hemin and oxidation of ABTS for chloroquine (correlation coefficient r = 0.99, p < 0.001). In contrast, no correlation was found for nitroindazoles despite these compounds reduced hemin crystallization. Neither for 8-hydroxyquinoline or β-carbolines that did not decrease crystallization of hemin nor promote oxidation. Peroxidation produced by free hemin in presence of chloroquine (100 µM) was diminished by antioxidants and peroxidase inhibitors such as trolox, ascorbic acid, catechin, hydroxylamine and sodium azide (Fig. 5a). Therefore, antioxidants protected against peroxidation catalyzed by free hemin allowed by quinoline drugs.

**Figure 6:**
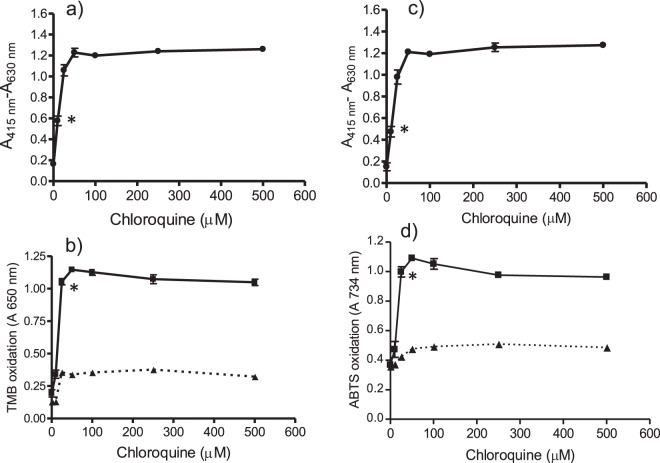
Hemin incubated with chloroquine (**a,c**) (●), and corresponding oxidation of TMB (**b**) or ABTS (**d**) in presence of globin (■). Incubation of hemin with chloroquine was carried out for 3 h and free hemin determined by absorbance (A_415 nm_-A_630 nm_). Oxidation of TMB was determined in presence of globin (10 µM) and H_2_O_2_ (100 µM) (**b**) and oxidation of ABTS in presence of globin (10 µM) and H_2_O_2_ 250 µM (**d**). Controls in absence of H_2_O_2_ (-▲-). Assays were in triplicate and data are significantly different from control without drug for this one and higher concentrations of drug (p < 0.01) (*).

Peroxidation catalyzed by free hemin build-up with drugs proceeds in presence of H_2_O_2_ at the pH of digestive vacuole. As proteins abound in digestive vacuole, peroxidation was evaluated under these conditions. Oxidation of TMB or ABTS by hemin solutions increased in presence of globin (Supplementary Fig. 5). In presence of globin, the oxidation of TMB or ABTS by free hemin accumulated during incubation was produced at much lower concentration of H_2_O_2_ (for TMB, 100 µM vs 1000 µM in absence of protein) (Fig. 6). Oxidation depended on the presence of both hemin and H_2_O_2_. A significant positive correlation was found among free hemin build-up (A_415 nm_-A_630 nm_) produced by chloroquine and the oxidation of TMB (r = 0.97, p < 0.01) or ABTS (r = 0.92, p < 0.01). The same qualitative trend of oxidation by hemin incubated with chloroquine was obtained in presence of BSA instead of globin (Supplementary Fig. 6). Therefore, free hemin build-up in the media by the action of quinoline drugs catalyzes peroxidative reactions that are enhanced in presence of proteins such as globin or BSA. Antioxidants and peroxidase inhibitors also decreased oxidation in presence of globin (Fig. 5b).

### Chloroquine inhibits cysteine proteases due to free hemin build-up

**Figure 7:**
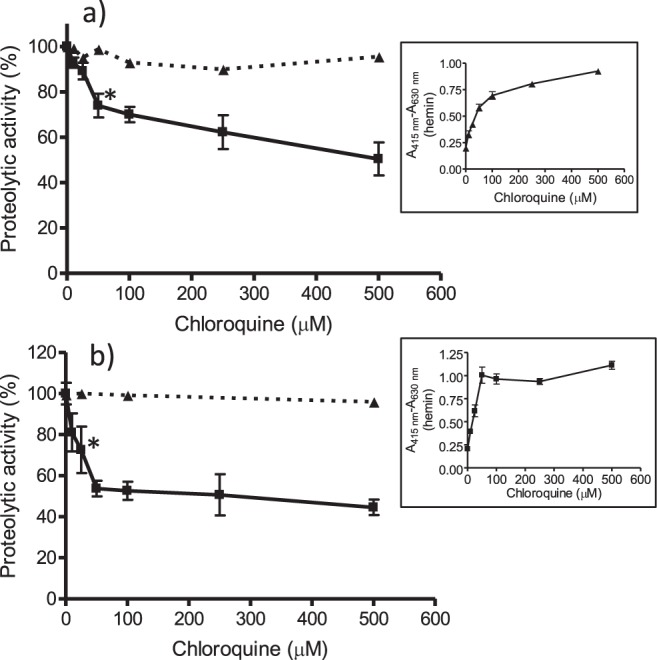
Inhibition of papain proteolytic activity by hemin incubated with chloroquine (3 h) (■). Proteolysis was measured as AMC released from Z-Phe-Arg-AMC from incubation media containing hemin plus chloroquine in presence of 1 mM H_2_O_2_ (**a**), or 75 µM H_2_O_2_ and globin (40 µM) (**b**). Controls (t = 0) without hemin (▲). Inserts correspond to hemin pattern during incubation (3 h). Basal proteolysis in absence of chloroquine was 21.22 ± 0.42 µM (**a**) or 46.41 ± 2.41 µM of AMC (**b**). Assays were in quadruplicate. (*) Data are significantly different (p < 0.01) from control without drug for this one and higher concentrations of drug.

**Figure 8:**
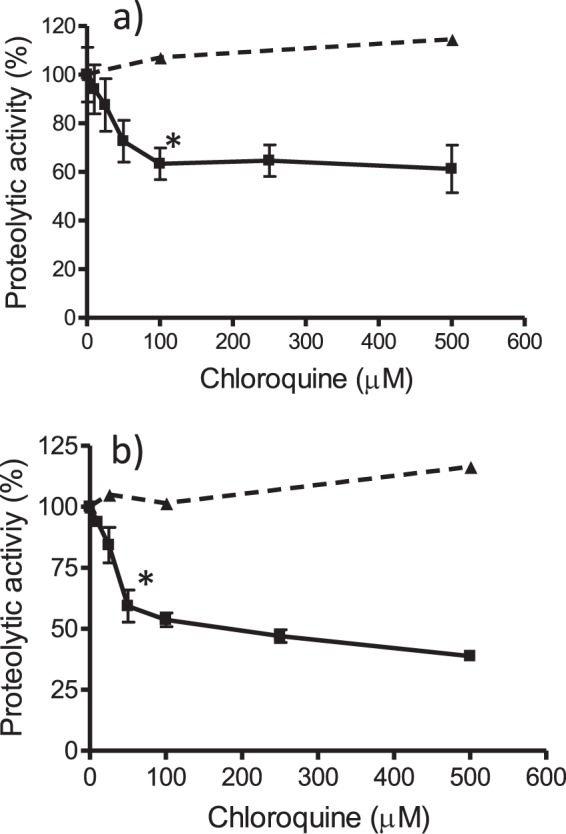
Inhibition of ficin (**a**) or cathepsin B (**b**) proteolytic activity by hemin incubated with chloroquine (3 h) (■). Proteolysis was measured as AMC released from Z-Phe-Arg-AMC in presence of globin (40 µM) and H_2_O_2_ (75 µM). Basal proteolysis in absence of drug was 24.0 ± 2.69 µM (**a**) and 28.9 ± 0.5 µM (**b**) of AMC. Assays with ficin and cathepsin were in quintuplicate and duplicate, respectively. Controls (t = 0) without hemin (▲) (*) Data are significantly different (p < 0.01) from controls without drug for this and higher concentrations of drug.

**Figure 9:**
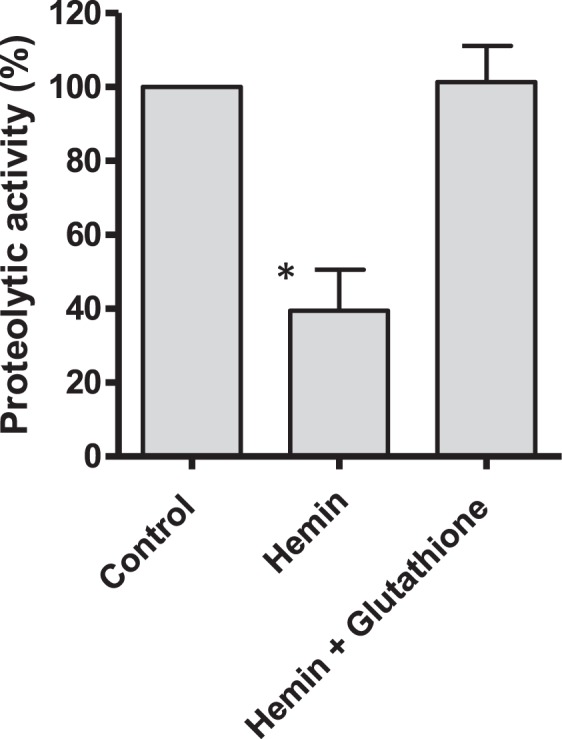
Inhibition of papain proteolytic activity by hemin incubated with chloroquine (100 µM). Proteolysis of Z-Phe-Arg-AMC was carried out as mentioned in experimental in presence of globin (40 µM) and H_2_O_2_ (75 µM), and with or without glutathione (500 µM). (*) Data are significantly different (p < 0.05) from control and from hemin plus glutathione.

Cysteine proteases metabolize hemoglobin in a process that is essential for growth and survival of parasite. The proteolytic activity of various cysteine proteases with incubation media of hemin with chloroquine was evaluated using Z-Phe-Arg-AMC (Supplementary Fig. 7). Proteolytic activity of papain, a model of cysteine protease that is similar to parasite´s falcipains, was inhibited up to a 50% by incubation media of hemin with increasing levels of chloroquine in assays containing H_2_O_2_ (Fig. 7a). Proteolytic activity was also inhibited in presence of globin at lower concentrations of H_2_O_2_ (Fig. 7b). The inhibition of papain correlated well with free hemin build-up by the action of chloroquine while chloroquine itself did not inhibit proteolysis. Other cysteine proteases such as ficin and cathepsin B were also inhibited in presence of increased concentrations of chloroquine (Fig. 8a,b) owing to enhanced levels of free hemin. Moreover, the same qualitative trend was obtained in presence of protein BSA instead of globin (Supplementary Fig. 8). These results show that free hemin build-up resulting from inhibition of β-hematin by quinoline drugs inhibits cysteine proteases in presence of H_2_O_2_. When glutathione was added no inhibition of proteolysis occurred (Fig. 9), suggesting that this antioxidant was able to reverse inhibition of proteolysis caused by hemin and H_2_O_2_.

## Discussion

Heme crystallization system in the digestive vacuole of *Plasmodium* has been a useful target for antimalarial drugs^16,49,50^. Chloroquine and other quinolines (Fig. 1) exert antimalarial actions by interfering with this system. These drugs accumulate into the acidic digestive vacuole reaching up to millimolar concentrations, and prevent heme sequestration resulting in toxicity^51^. The biochemical mechanisms underlying these processes are still poorly understood despite their importance for the design of novel and more efficient drugs against resistant parasites^52^. *Plasmodium* detoxifies heme through its conversion to insoluble crystalline ferriprotoporphyrin IX dimer called hemozoin (β-hematin). This process may occur by self-assembly (autocatalytic) near lipid/water surfaces^30,37,53,54^, or be catalyzed by specific heme detoxification proteins^24,55^. Drugs targeting this process have been screened on the basis of differential solubilization of β-hematin and hemin^27,56^. These assays are often troubled by the formation of aggregates distinct from β-hematin. A spectrophotometric assay was used here to assess the contribution of free hemin and β-hematin^27,47,57^. In this assay, active quinolines inhibited β-hematin formation and proportionally increased free hemin. Chloroquine, quinacrine and amodiaquine were the most active drugs whereas quinidine, quinine and mefloquine had lower potency. Two nitroindazoles had activity comparable to chloroquine and quinacrine whereas 8-hydroxyquinoline and β-carbolines were inactive. It is generally assumed that active quinoline drugs (Fig. 1) interact with free hemin and block hemozoin synthesis. The incorporation of quinoline-heme complexes into the growing crystal of hemozoin helps to terminate the process of crystallization of hemin^35,58^. Results obtained herein and elsewhere suggest that drugs with protonated nitrogen and an aliphatic chain with a tertiary nitrogen have higher activity whereas the pyridine nitrogen has less effect^33,37^. The electron rich planar area of quinoline interacts with hemin whereas basic nitrogen interacts with anionic sites^33,59^. These quinoline-heme interactions show molecular diversity and quinolines may act differentially with various forms of heme (monomer vs dimer) and perturb equilibrium between them playing a role in the inhibition of β-hematin^60,61^. β-Carbolines are quinoline analogs with reported antimalarial activity^44,62^. Inhibition of hemozoin and intercalation of DNA have been proposed as possible mechanisms of action for β-carbolines. Results obtained here show that these substances do not inhibit β-hematin suggesting they may work under other mechanisms^63^.

Active quinolines increased free hemin that catalyzed peroxidation reactions under conditions occurring in digestive vacuole. A significant correlation was found between the free hemin increased due to the action of antimalarial drugs and peroxidation (oxidative effects). Then, active quinolines may ultimately increase oxidative effects in the parasite through increased free hemin. The most active pro-oxidant drugs were by this order: chloroquine, quinacrine, amodiaquine, quinine, quinidine and mefloquine. 8-Hydroxyquinoline and β-carbolines were unable to increase oxidation as they lacked inhibitory effects against β-hematin and consequently did not increase free hemin. The occurrence of phenolic substituents in the quinoline structure (i.e. 8-hydroxyquinoline) decreased oxidation catalyzed by hemin likely due to antioxidant properties (Fig. 4). Nitroindazoles (DIM 5 and DIM 32) did not promote oxidation because they precipitated hemin that was not available for peroxidative reactions. However, this hemin contributed to absorbance as it redissolved during assay. Thus, differences between assays of hemin/β-hematin by absorbance and oxidation could detect possible aggregates of hemin different from β-hematin as occurred here with nitroindazoles.

Increased peroxidation by quinoline drugs was evidenced in presence of H_2_O_2_ and depended on hemin. Reactive oxygen species (ROS) could participate in the mechanism of action of quinoline drugs (*i.e*. chloroquine). Indeed, *Plasmodium* brakes down high levels of hemoglobin to peptides and amino acids. During this process free heme (Fe^2+^) converts into hemin (Fe^3+^) with subsequent production of superoxide anion (O_2_^●−^) that under acidic conditions dismutates to H_2_O_2_^64^. It is estimated that up to 15 mM H_2_O_2_ could be produced within digestive vacuole, exposing parasites to high fluxes of ROS^42,65^. The activity of chloroquine against *P. falciparum* is enhanced in presence of H_2_O_2_^33,66,67^ whereas hemin increases *in vivo* after treatment with chloroquine^68^. As shown here, those high levels of H_2_O_2_ could be involved in peroxidase reactions catalyzed by the increased levels of free hemin allowed by quinoline drugs. Interestingly, proteins abound in the digestive vacuole and this work has shown that peroxidation catalyzed by free hemin highly increased in presence of globin resulting in lower concentrations of H_2_O_2_ needed for oxidation. Therefore, the digestive vacuole contains enough H_2_O_2_ with high levels of proteins such as globin, that could result in increased oxidative effects (oxidative stress) triggered by soluble free hemin allowed by antimalarial drugs (chloroquine). Previously, it has been suggested that chloroquine could act through oxidative stress because it may host highly reactive radicals^69^. Results in this work, however, suggest that active quinolines contribute to oxidative stress thorough an increase of free hemin but they have no pro-oxidant effect by themselves. Thus, peroxidative actions depended on the presence of soluble free hemin in incubation media although this hemin might be in different forms and as hemin-quinoline complexes^61^ that keep peroxidative actions. Quinoline drugs which accumulate into the parasite digestive vacuole increase soluble free hemin which may trigger peroxidation and oxidative stress supporting the assumption that accumulation of free hemin may result in oxidative damage to lipid membranes, proteins, and DNA with subsequent toxicity for the parasite^6,42,70^. Resistance to quinolines may arise from a decrease in drug level at the site of action and/or mechanisms of defense against heme^16,33,41,71^. Antioxidants and peroxidase ligands (trolox, catechin, ascorbic acid, sodium azide and hydroxylamine) inhibited peroxidative actions of hemin. The antioxidant system of parasite could reduce peroxidase actions of hemin and decrease efficacy of quinolines. In this regard, drugs that increase hemin accumulation while decreasing antioxidant effects might be most useful to increase antimalarial activity.

Inhibition of proteolysis is a current target for new antimalarial drugs^4,15,72^. A number of protease inhibitors are active against *Plasmodium*^5,73^. Numerous cysteine proteases have been identified in the genome of *Plasmodium*. They are involved in essential processes for parasite survival and growth such hemoglobin digestion, erythrocyte rupture and host cell invasion^5,73^. *Plasmodium* consumes large quantities of hemoglobin from host cell. Once inside the parasite, hemoglobin is shuttled to digestive vacuole where it is digested by successive action of aspartate and cysteine proteases such as papain-like falcipains^5^. This process results in high levels of globin, peptides and amino acids that are transported into cytoplasm and used for cell growth^15^. Antimalarial drugs could interfere with hemoglobin digestion during blood stages of malarial life-cycle^33,68,74^. Chloroquine inhibits lysosomal functions^34^ and protein degradation in trophozoite-infected erythrocytes^75^. The inhibition of lysosomal function may lead to parasite starvation^76,77^ and vacuole abnormalities^15^. Results in this work have shown that free hemin enhanced by the action of chloroquine was able to inhibit cysteine proteases. This was evidenced for three distinct enzymes: papain, ficin and cathepsin B. Papain is similar to falcipain in *Plasmodium* whereas cathepsin B participates in the degradation of hemoglobin in *S. mansoni*^78,79^. Inhibition was due to free hemin (or hemin complexes) allowed by chloroquine but not to chloroquine itself. It occurred in presence of H_2_O_2_ suggesting a likely involvement of peroxidative reactions that increased in presence of proteins such as globin. Cysteine proteases contain an essential sulfhydryl group sensitive to oxidation and inactivation due to the formation of mixed disulfides^80,81^, or sulfenic acid (R-SOH) that are reversible in presence of glutathione^82^. Here, the inhibition of proteolysis by cysteine proteases due to free hemin allowed by chloroquine was reversed with glutathione. Previously, chloroquine response to *Plasmodium berguei*, a rodent malaria species, was affected by glutathione levels^67^. Then, inactivation of cysteine proteases by quinoline drugs might occur under conditions of oxidative stress in a deficit of glutathione. Then, the antioxidant system of parasite could contribute to reduce inhibition of proteolysis triggered by chloroquine.

**Figure 10:**
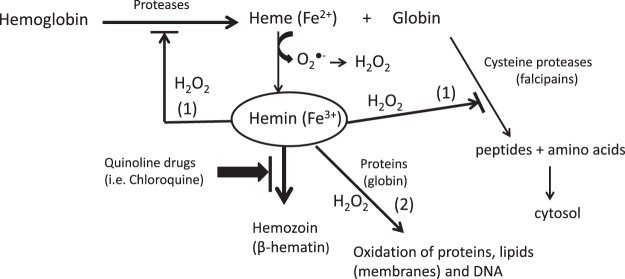
Mechanism of action of quinoline drugs inhibiting hemozoin in the digestive vacuole of *Plasmodium* inside erythrocytes or in other organisms that use hemoglobin as food. This inhibition enhances the levels of free hemin which promote peroxidase reactions with H_2_O_2_ arising from heme (Fe^2+^) oxidation. Hemin and peroxidase reactions inhibit cysteine proteases needed for degradation of proteins used for parasite growth (1), and may also oxidize proteins (enzymes), lipids and DNA resulting in parasite damage (2).

Beyond *Plasmodium*, heme aggregation is an efficient mechanism of detoxification in other organisms that use hemoglobin such as *S. mansoni* or *R. prolixus*^23,64,83,84^. Chloroquine increases free hemin in the midgut of *R. prolixus*  resulting in lipid peroxidation^85^. Blood feeding organisms including *Plasmodium* contain proteases that break down hemoglobin affording heme and also globin, peptides and amino acids that are used for growth and survival (Fig. 10). Heme is oxidized to hemin (Fe^3+^) releasing H_2_O_2_. The former organisms detoxify hemin by its conversion into non-toxic hemozoin. However, in presence of active quinolines (e.g. chloroquine or quinacrine), hemin may remain free in the vacuole to interact with globin or other proteins, and can be oxidized by H_2_O_2_ to higher oxidative iron forms (oxoferryl iron (Fe(IV) = O) or oxoferryl porphyrin π-cation radicals $[({{\rm{P}}}^{\bullet +}){{\rm{Fe}}}^{{\rm{IV}}}={\rm{O}}]$) which are highly oxidant species resembling compound I of heme peroxidases)^86–88^. These species can oxidize small molecules and metabolites, proteins, lipid membranes, and DNA resulting in parasite damage. Under these conditions free hemin may also inactivate enzymes such as papain-like cysteine proteases that are essential for parasite survival and growth.

Taken together results obtained in this study shed light on the mechanisms of toxicity of antimalarial quinoline drugs in blood feeding organisms. However, these results and evidences should be tested further and confirmed *in vivo* in the own malaria parasite where complexity increases substantially and the actual conditions could differ (*e.g*. H_2_O_2_ concentrations in digestive vacuole, specific cysteine proteases involved, antioxidant mechanisms, among many other factors), and these changes might affect quinoline-heme interactions as well as peroxidative actions and inhibition of proteolysis.

In conclusion, results show that active quinoline drugs inhibit β-hematin resulting in increased levels of free hemin. Under conditions expected in the parasite digestive vacuole this free hemin catalyzes oxidative reactions and inhibition of cysteine proteases. Peroxidation and inhibition of proteolysis due to free hemin was enhanced in presence of globin. Results evidenced a positive correlation between quinoline drugs, free hemin increase, and enhanced peroxidation and inhibition of proteolysis. These mechanisms suggest how these drugs may work against malaria parasite. Thus, anti-parasitic drugs that increase free hemin could trigger oxidative stress through peroxidase reactions resulting in inhibition of proteolysis and damage to macromolecules and membranes causing toxicity and parasite death. Oxidation promoted by chloroquine was inhibited by antioxidants and peroxidase inhibitors and the inhibition of proteolysis blocked by glutathione. Therefore, the antioxidant system of parasite could contribute to quinoline drug resistance. These results could be useful for the design of more powerful antimalarial drugs.

## Material and Methods

Hemin chloride, chloroquine, quinacrine, amodiaquine, mefloquine, 8-hydroxyquinoline, quinine, quinidine and the β-carbolines norharman, tryptoline, harman, and harmine were obtained from Sigma-Aldrich. Indazole compounds 1,1´-[2,2´-biphenyldiyl)bismethylene]bis(5-nitro-1*H*-indazol-3-ol) (DIM-32) and 1,1´-(*o*-xylylene)bis(5-nitro-1*H*-indazol-3-ol) (DIM-5) were previously synthetized^45^. 3,3′,5,5′-Tetramethylbenzidine (TMB), 2,2′-azinobis(3-ethylbenzthiazoline-6-sulphonic acid) (ABTS) and tween 20 were obtained from Sigma and cysteine from Merck. DMSO and hydrogen peroxide (H_2_O_2_) were from Scharlau and Panreac, respectively. Globin was obtained from bovine hemoglobin (Sigma) by precipitation in acetone-0.1% HCl at low temperature^89,90^, and lab-stored crystallized and lyophilized bovine serum albumin (BSA) was from Sigma. Cysteine proteases: papain from *Carica papaya*, ficin from fig tree latex and cathepsin B from bovine spleen were obtained from Sigma. The peptide Z-Phe-Arg-AMC was purchased from Bachem, and 7-amino-4-methylcoumarin (AMC) from Sigma.

### Crystallization of hemin to β-hematin and inhibition by quinoline drugs

Eppendorf tubes (1 mL, final volume) containing by order: hemin chloride dissolved in DMSO (8 µL to reach 0.036 mg/mL, final conc.), quinoline or β-carboline drug dissolved in water (0–500 μM, final conc.), or nitroindazole dissolved in DMSO (0–100 µM, final conc.), freshly prepared detergent tween 20 dissolved in water (15 µL to reach 0.015 mg/mL, final conc.), and 100 mM acetate buffer (pH 4.8) to reach 1 mL, were vortexed, and incubated in the dark at 37 °C for 2 h at 700 rpm using a thermo shaker (Biosan TS100). Then, the tubes were slightly moved and kept in the dark for 1 h at room temperature to continue with precipitation of crystallized hemin (β-hematin). During the assay free hemin disappears from the media as it is converted to β-hematin that precipitates. Control assays without incubation were also carried out. After the incubation period, an aliquot (400 µL) of supernatant was used for studying peroxidation as mentioned below. With the rest (600 µL) a colorimetric method was used to determine the effect of drugs on hemin crystallization. Colorimetric methods afford comparable results to those of purification of β-hematin^47,91,92^. For this, the 600 µL were pippeted three times, and absorbance measured first at 630 nm and then at 415 nm against a blank of 100 mM acetate buffer (pH 4.8). Any drug that inhibits the synthesis of β-hematin will increase free hemin, so that its activity as inhibitor of hemin crystallization can be determined. The concentration of hemin was calculated from a calibration curve of absorbance (A_415 nm_-A_630 nm_) vs hemin standard. To A_415 nm_-A_630 nm_ values from incubation media containing the drug were subtracted those without drug, and divided by A_415 nm_-A_630 nm_ with drug without incubation (control at t = 0). It affords a measure of remaining free hemin (%) that correlates with inhibition of β-hematin. IC_50_ values (concentration of drug that inhibits a 50% the formation of β-hematin or that increases free hemin a 50%) and the highest percentage of inhibition of hemin crystallization (or maximum % of free hemin achieved) were determined. The IC_50_ values were calculated from at least five different incubation experiments for each compound (0–500 µM concentration range) using GraphPad Prism 4.0. Controls with drugs without the incubation period were also prepared. DMSO used as solvent for indazole compounds had no effect up to 15%. To study the fate of hemin during assay, UV-VIS spectra of solutions of hemin and hemin plus chloroquine (100 µM) were monitored during incubation time (37 °C, 700 rpm).

### Isolation and characterization of β-hematin

Forty eppendorf tubes (1 mL, final volume) containing hemin dissolved in DMSO (0.036 mg/mL, final conc.), freshly prepared tween 20 (final conc., 0.015 mg/mL), and 995 µl 100 mM acetate buffer (pH 4.8) were vortexed and incubated in the dark at 37 °C for 2 h while shaking at 700 rpm using a thermo shaker. The tubes were kept at room temperature in the dark for 1 hour and then centrifuged at 10000 rpm for 10 min. The precipitate was washed with buffer, centrifuged again and washed two additional times with water. The precipitate of β-hematin, a black powder, was dried and characterized by IR spectrometry.

### Peroxidation activity of hemin and activity of quinoline drugs

Incubations of hemin with quinoline drugs were carried out as mentioned above and used to study peroxidation as follows:

#### Oxidation of peroxidase substrates TMB and ABTS

Incubation media containing hemin, quinoline drugs and tween 20 in acetate buffer (pH 4.8) were prepared as above and incubated for 2 h at 37 °C (700 rpm) and kept 1 h at room temperature. Controls without incubation period were also prepared. An aliquot (400 µL) of supernatant was added to 100 mM acetate buffer (pH 4.8) (106 µL), 10 mM H_2_O_2_ (60 µL) (1 mM, final conc.) and 8.83 mM 3,3′,5,5′-tetramethylbenzidine (TMB) disolved in methanol (34 µL) (500 µM, final conc.) to reach 600 µL (final volume). The tubes were vortexed and incubated in the dark for 20 min at 37 °C in a water bath. An aliquot (500 µL) was used to determine oxidation of TMB by absorbance measured at 650 nm (blue charge-transfer complex from TMB^●+^ radical cation), and then at 450 nm following addition of 2 N HCl (250 µL) (yellow TMB diimine) using acetate buffer as a blank^93,94^. Oxidation of TMB was confirmed by measuring the corresponding UV-VIS absorption spectra. Oxidations with hemin and hemin plus drug were also determined at time zero (absence of incubation). The concentration of drug needed to reach a 50% of the initial oxidation of TMB taken as 100% was calculated as EC_50_, and values calculated from at least five different incubation experiments. In other parallel experiments, an aliquot (400 µL) of supernatant from incubations of hemin plus quinoline drugs (2 + 1 h) or from controls without incubation, was added with methanol (110 µL), 10 mM H_2_O_2_ solution (60 µL) (1 mM, final conc.) and 10 mM ABTS solution in water (30 µL) (500 µM, final conc.), and tubes incubated at 37 °C for 10 min in the dark. Absorbance at 734 nm was measured to determine oxidation of ABTS (ABTS^+•^ cation radical)^95^. Oxidation of ABTS was confirmed with the corresponding UV-VIS absorption spectra. The concentration of drug needed to reach a 50% of the initial oxidation of ABTS that was taken as 100% was calculated as EC_50_ and values calculated from at least five different incubation experiments. Control assays were also carried out in order to determine the influence of drugs, hemin and H_2_O_2_ in the oxidation of TMB and ABTS substrates.

#### Oxidation of TMB and ABTS in presence of globin or BSA

Eppendorf tubes (1 mL, final volume) containing by order: hemin dissolved in DMSO (0.036 mg/mL), chloroquine (0–500 µM), freshly prepared tween 20 (0.015 mg/mL) and acetate buffer (pH 4.8) 100 mM, were vortexed and incubated as above at 37 °C for 2 h in the dark in a shaker at 700 rpm and kept at room temperature for 1 h. Controls without incubation period were also prepared. An aliquot of 400 µL was taken from supernatant and added to globin (10 µM, final concentration) or BSA (20 µM, final concentration) solutions, H_2_O_2_ (100 µM, final concentration) and 3,3′,5,5′-tetramethylbenzidine (TMB) disolved in methanol (500 µM, final concentration) to reach 600 µL (final volume), and vortexed and incubated in the dark for 20 min at 37 °C in a water bath. An aliquot of 500 µL was used to determine oxidation of TMB by absorbance at 650 nm (charge-transfer complex) and 450 nm after addition of 2 N HCl (250 µL) (TMB diimine). In other parallel experiments, an aliquot of supernatant (400 µL) taken from samples containing hemin and drug after incubation (2 + 1 h) or from controls without incubation was added to globin solution (10 µM, final conc.), H_2_O_2_ solution (250 µM, final conc.) and ABTS solution in water (500 µM, final concentration), and incubated at 37 °C for 10 min in the dark. Then, absorbance at 734 nm was measured to determine oxidation products of ABTS (ABTS^+•^ cation radical)^95^. Controls in absence of H_2_O_2_ or hemin were also measured.

To determine the effects of antioxidants and peroxidase inhibitors on the oxidation of TMB, incubation media of hemin with chloroquine (100 µM) were carried out as above (2 + 1 h), and oxidation of TMB was carried out in presence of catechin (1 mM), trolox (1 mM), ascorbic acid (1 mM), sodium azide (1 mM) or hydroxylamine (1 mM) in absence or in presence of proteins as mentioned above.

### Proteolytic activity of cysteine proteases and effects of chloroquine

Incubations of hemin with chloroquine were used to investigate possible effects on proteolysis. Proteolysis was studied with three distinct cysteine proteases: papain, ficin and cathepsin-B using Z-Phe-Arg-AMC peptide. Incubation of hemin with or without chloroquine was carried out as above. Eppendorf tubes (1 mL, final volume) containing by order: hemin dissolved in DMSO (0.036 mg/mL), detergent tween 20 (0.015 mg/mL), chloroquine from 0–500 µM and 100 mM acetate buffer (pH 4.8) were vortexed and incubated for 2 h at 37 °C at 700 rpm and kept for 1 h at room temperature. An aliquot of 600 µL was taken to study proteolysis as mentioned below. The rest (400 µL) was mixed by pippeting three times, and absorbance measured at 630 nm and 415 nm to determine hemin as above. Proteolysis was studied as follows: a) An aliquot (600 µL) from incubation media (2 + 1 h) was added with 100 mM acetate buffer (pH 4.8), papain (100 µg prot./mL with 3.5 µM cysteine) and H_2_O_2_ (1 mM). The tubes were pre-incubated for 5 min at 37 °C and added with Z-Phe-Arg-AMC (100 µM) (final volume of mixture, 800 µL). After 20 min at 37 °C, reaction was stopped with 2 N HCl:MeOH (1:1) (80 µL), and samples analyzed by RP-HPLC to determine proteolysis as AMC released (rate of hydrolysis as µM AMC released/min). Assays were made in quadruplicate. b) Aliquots (600 µL) from incubation media (2 + 1 h) were added with 100 mM acetate buffer (pH 4.8), globin (40 µM), papain (20 µg prot./mL with 2.5 µM cysteine), and H_2_O_2_ (75 µM). The samples were pre-incubated for 5 min at 37 °C and added with Z-Phe-Arg-AMC (100 µM) (final volume of mixture, 800 µL). After 5 min at 37 °C, the reaction was stopped with 2 N HCl:MeOH (1:1) (80 µL), and samples vortexed and injected into HPLC for analysis of AMC. The assays were made in quadruplicate. c) Aliquots (600 µL) from incubation media (2 + 1 h) of hemin with chloroquine were added with 100 mM acetate buffer, BSA (60 µM), papain (20 µg prot./mL with 2.5 µM cysteine), and H_2_O_2_ (75 µM). The samples were pre-incubated for 5 min at 37 °C and added with Z-Phe-Arg-AMC (100 µM) (final volume of mixture, 800 µL). After 5 min at 37 °C, the reaction was stopped with 2 N HCl:MeOH (1:1) (80 µL), and samples vortexed and injected into HPLC for analysis of AMC. The assays were made in sextuplicate. d) Aliquots (600 µL) from incubation media of hemin with chloroquine (2 + 1 h) were added with 100 mM acetate buffer, globin (40 µM), ficin (100 µg prot/mL with 2.5 µM cysteine), and H_2_O_2_ (75 µM). The samples were pre-incubated for 5 min at 37 °C and added with Z-Phe-Arg-AMC (100 µM) (final volume of mixture, 800 µL). After 5 min at 37 °C, the reaction was stopped with 2 N HCl:MeOH (1:1) (80 µL), and samples injected into HPLC for analysis of AMC. Assays were carried out in quintuplicate. e) Aliquots of 150 µL of incubation media (2 + 1 h) of hemin with chloroquine were added with globin (40 µM), cathepsin B (21.75 µg prot./mL protein with 2.5 µM cysteine) and H_2_O_2_ (75 µM) and pre-incubated 5 min at 37 °C; then added with Z-Phe-Arg-AMC (100 µM) (final volume, 200 µL) and incubated 10 min at 37 °C. The reaction was stopped with 2 N HCl:methanol (1:1) (20 µL), vortexed and injected into the HPLC to determine AMC released into the assays. Assays were carried out in duplicate. The proteolytic activity by papain, ficin or cathepsin B in incubation media of hemin plus chloroquine was calculated as a percentage of initial proteolysis without drug taken as 100%. The effect of glutathione on proteolysis with papain was determined as in protocol b) from incubation media of hemin plus chloroquine (100 µM) with globin (40 µM), H_2_O_2_ (75 µM) and glutathione (500 µM).

### RP-HPLC analysis of AMC and Z-Phe-Arg-AMC

Samples (20 µL) of proteolysis assays were injected into a 1050 HPLC equipped with a DAD and a 1046 fluorescence detector. The chromatographic column was a 150 × 3.8 mm, 4 µm Novapak-C18 (Waters); Chromatographic conditions were: ammonium phosphate buffer (pH 3) (eluent A), and 20% eluent A in ACN (eluent B); the gradient was at 8 min 32% B and at 18 min 90% B. Detection was carried out with a DAD at 320 and 355 nm and fluorescence at 352 nm excitation and 442 nm emission. AMC released from Z-Phe-Arg-AMC peptide by cysteine proteases was analyzed at 355 nm and concentration obtained by using a calibration curve with AMC standard.

### Spectrophotometric (UV-VIS) and infrared (IR) spectra

Absorption spectra from incubation media containing hemin and hemin plus chloroquine as well as from samples of oxidation of TMB and ABTS substrates were measured with a spectrophotometer Beckman Coulter Du 80 at 37 °C. To obtain IR spectra, potassium bromide pellets were prepared from dried samples of β-hematin obtained as mentioned above, and spectra were acquired with an IR Perkin Elmer 681 spectrophotometer.

### Data and statistical analysis

The inhibition of hemozoin (%) and oxidation of TMB or ABTS substrates (%) as a function of quinoline drugs incubated with hemin were fit to non-linear regression curves to calculate IC_50_ or EC_50_ values using GraphPad Prism, 4.0. Correlation studies and statistical analysis were carried out and significance assumed for p < 0.05.

## Supplementary information

 Supplementary Information

## Data Availability

Data generated and analyzed during this study are included in the published article (and its supplementary information files). Materials and data are also available from corresponding author on request.
