# Supplementary material for: Antimalarial Quinoline Drugs Inhibit β-Hematin and Increase Free Hemin Catalyzing Peroxidative Reactions and Inhibition of Cysteine Proteases
Source: Sci Rep. 2019 Oct 28;9:15398. doi: 10.1038/s41598-019-51604-z (PMC6817881; doi:10.1038/s41598-019-51604-z)
Supplement: Supplementary file 1 — Supplementary Information [file 41598_2019_51604_MOESM1_ESM.pdf]

SUPPLEMENTARY MATERIAL

ANTIMALARIAL QUINOLINE DRUGS INHIBIT β-HEMATIN AND INCREASE FREE HEMIN CATALYZING PEROXIDATIVE REACTIONS AND INHIBITION OF CYSTEINE PROTEASES

Tomás Herraiz[1*], Hugo Guillén[1], Diana González-Peña[1], Vicente J. Arán[2].

[1]Instituto de Ciencia y Tecnología de Alimentos y Nutrición (ICTAN). Spanish National Research Council (CSIC), Juan de la Cierva 3, 28006, Madrid (Spain).
[2] Instituto de Química Médica (IQM-CSIC). Juan de la Cierva 3, 28006, Madrid (Spain).

Supplementary material Figure legends

Supplementary Figure 1. Infrared spectrum (IR) of β-hematin formed during incubation of hemin in presence of tween 20 at pH 4.8 and 37ºC.

Supplementary Figure 2. Photography of tubes containing hemin incubated with increasing concentrations of chloroquine (0-500 µM) in presence of tween 20 at pH 4.8 for 2h (700 rpm) at 37ºC followed by 1h at room temperature.

Supplementary Figure 3. Evolution of hemin (%) (a) and oxidation of TMB (b) during incubation of hemin at 37ºC (700 rpm) with (▲) or without (■) chloroquine (100 µM). Assays were in duplicate. (*) Data are significantly different from control (t=0 h) for this one and higher incubation time ($p<0.01$).

Supplementary Figure 4. Oxidation of TMB (Absorbance at 650 nm) (a) and oxidation of ABTS (Absorbance at 734 nm) (b), as a function of concentration of hemin with (■) or without (▼) H_2O_2 (1 mM). Assays were in duplicate.

Incubations were carried out at 37ºC for 20 min or 10 min for TMB (500 µM) or ABTS (500 µM), respectively.

Supplementary Figure 5. Oxidation catalyzed by hemin of TMB (A 650 nm) (a) and ABTS (A 734 nm) (b) in presence of increasing concentrations of globin (■). Control in absence of hemin (▼). Results are from duplicates. For oxidation of TMB, samples with hemin (0.036 mg/mL) and chloroquine (100 µM) were added with globin, H_2O_2 (100 µM) and TMB (500 µM) and incubated (37ºC, 20 min); for oxidation of ABTS, samples with hemin (0.036 mg/mL) and chloroquine (100 µM) were added with globin, H_2O_2 (250 µM) and ABTS (500 µM) and incubated (37ºC, 10 min).

Supplementary Figure 6. Increase of free hemin with concentration of chloroquine (above), and corresponding oxidation of TMB in presence of BSA (20 µM) and H_2O_2 (100 µM) (below). Incubation of hemin and chloroquine was carried out for 3h as mentioned in experimental. Control in absence of H_2O_2 (-▲-**).** Data are from six assays. (*) Data are significantly different for this one and higher concentrations of drug ($p<0.01$).

Supplementary Figure 7. Typical RP-HPLC chromatogram of Z-Phe-Arg-AMC proteolysis by papain in presence of hemin incubated (3h) in absence (a) or in presence of chloroquine (500 µM) (b). Assay of proteolysis was carried out as indicated in experimental with papain, globin (40 µM) and H_2O_2 (75 µM). Detection of AMC is carried out at 355 nm.

Supplementary Figure 8. Increase of free hemin (above), and inhibition of papain proteolytic activity (below) by hemin incubated with chloroquine (3h). Proteolysis was studied as indicated in experimental from incubation media containing hemin plus chloroquine in presence of BSA (60 µM) and H_2O_2 (75 µM). Results are from six assays. (*) Data are significantly different from control without drug for this one and higher concentrations of drug ($p<0.01$).

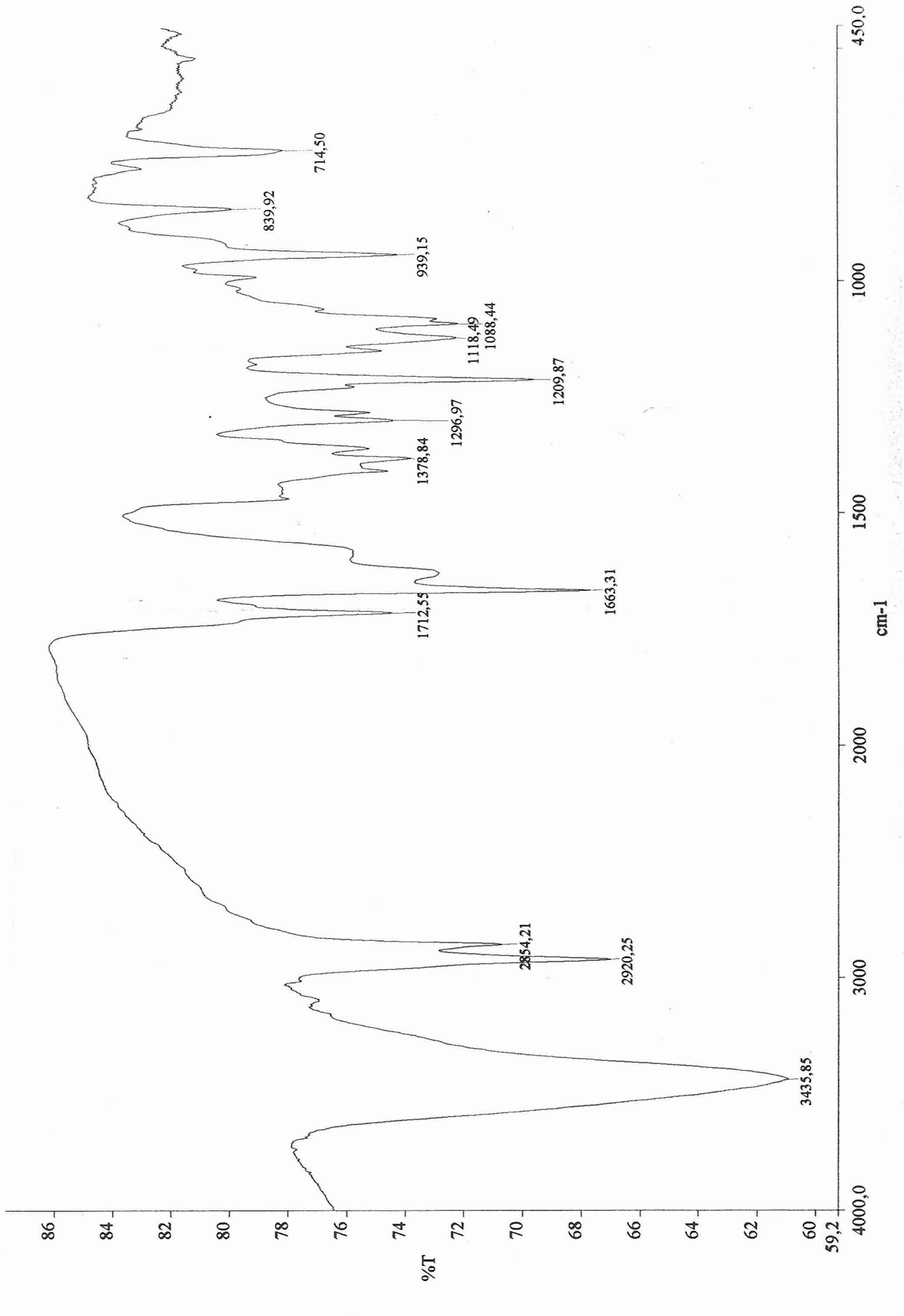

Supplementary Figure 1

Supp. Fig.2

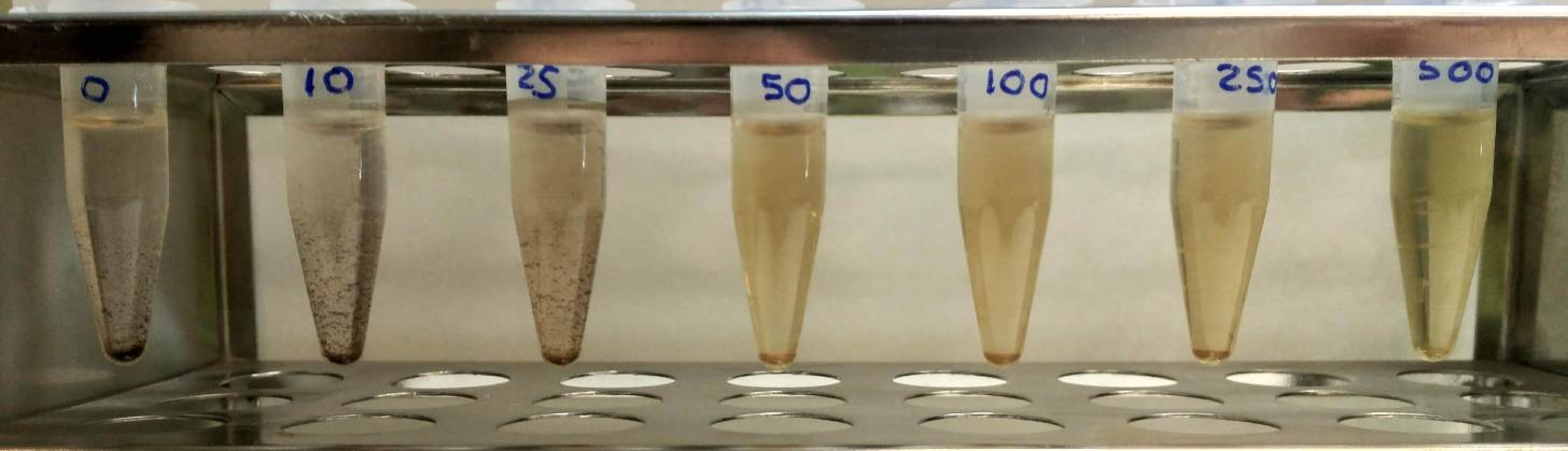

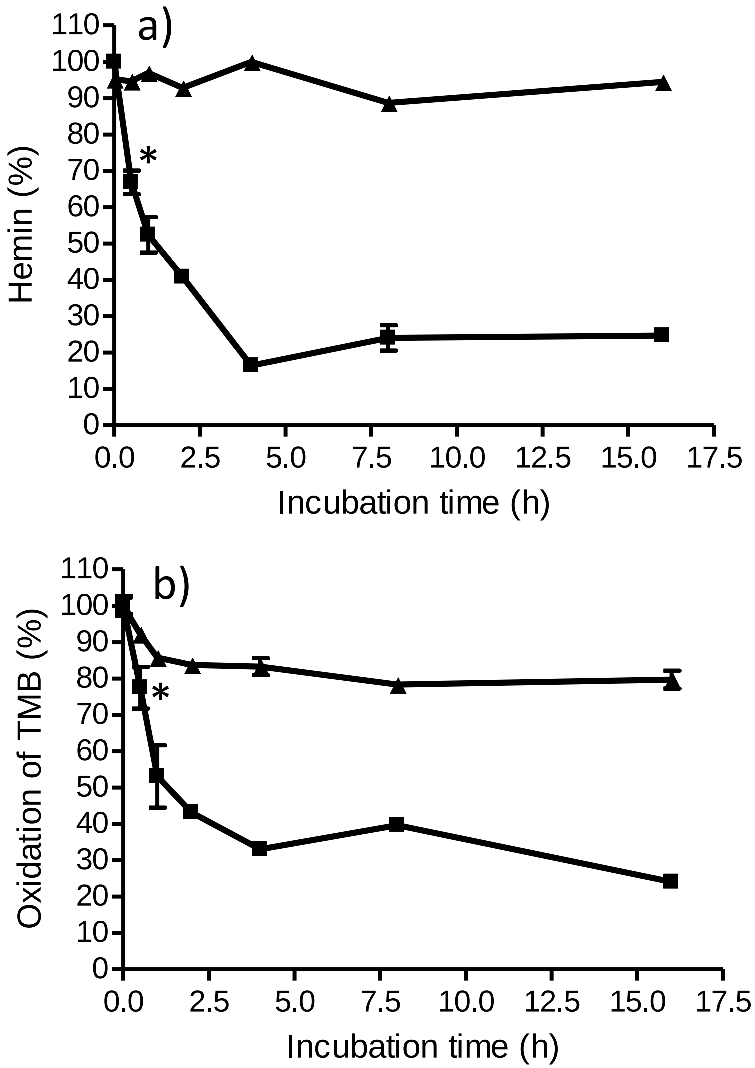

Supplementary Figure 3

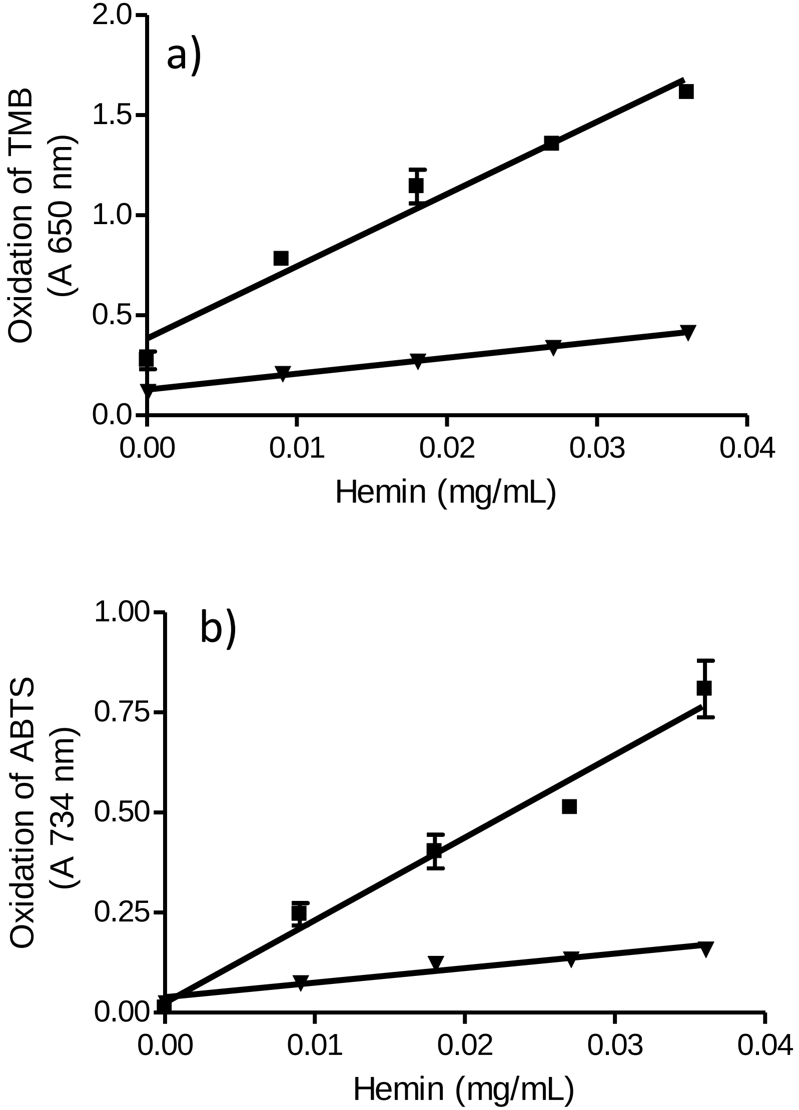

Supplementary Figure 4

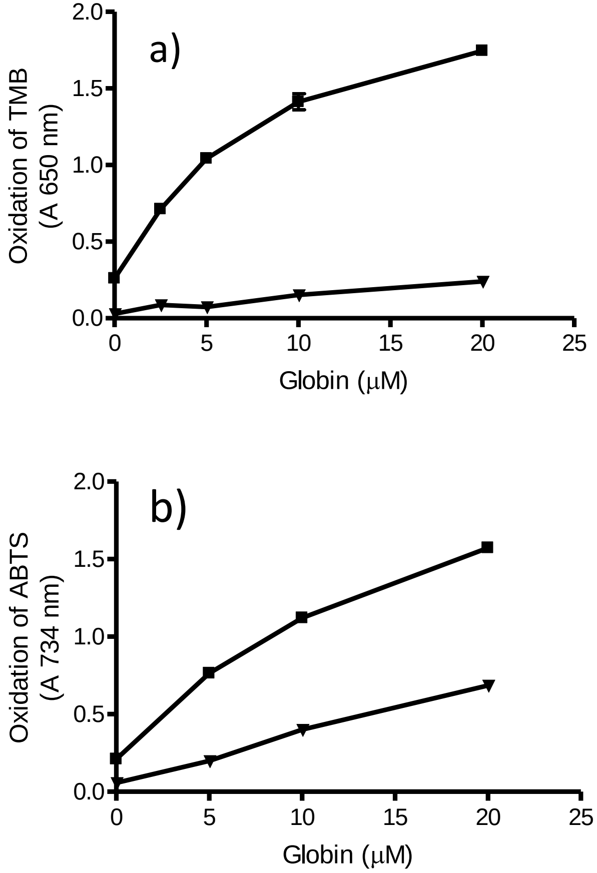

Supplementary Figure 5

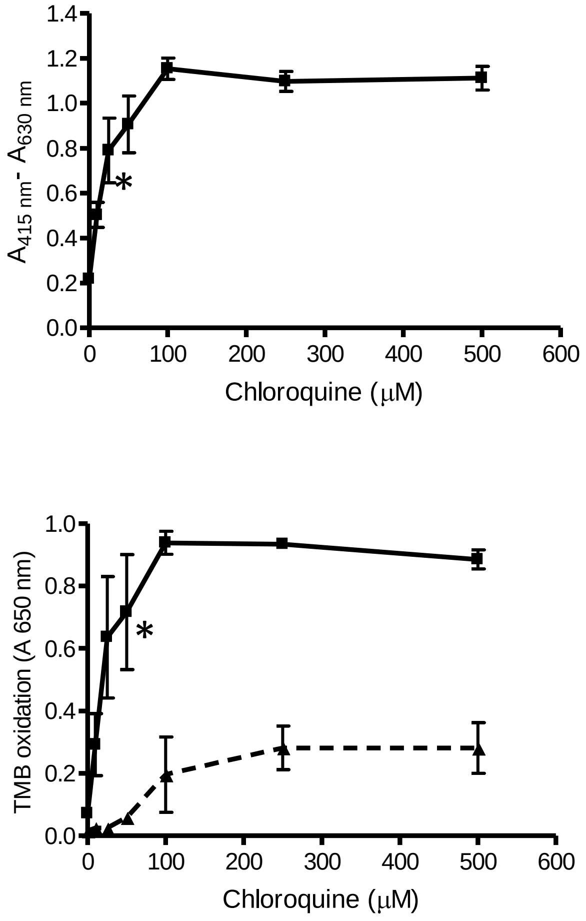

Supplementary Figure 6

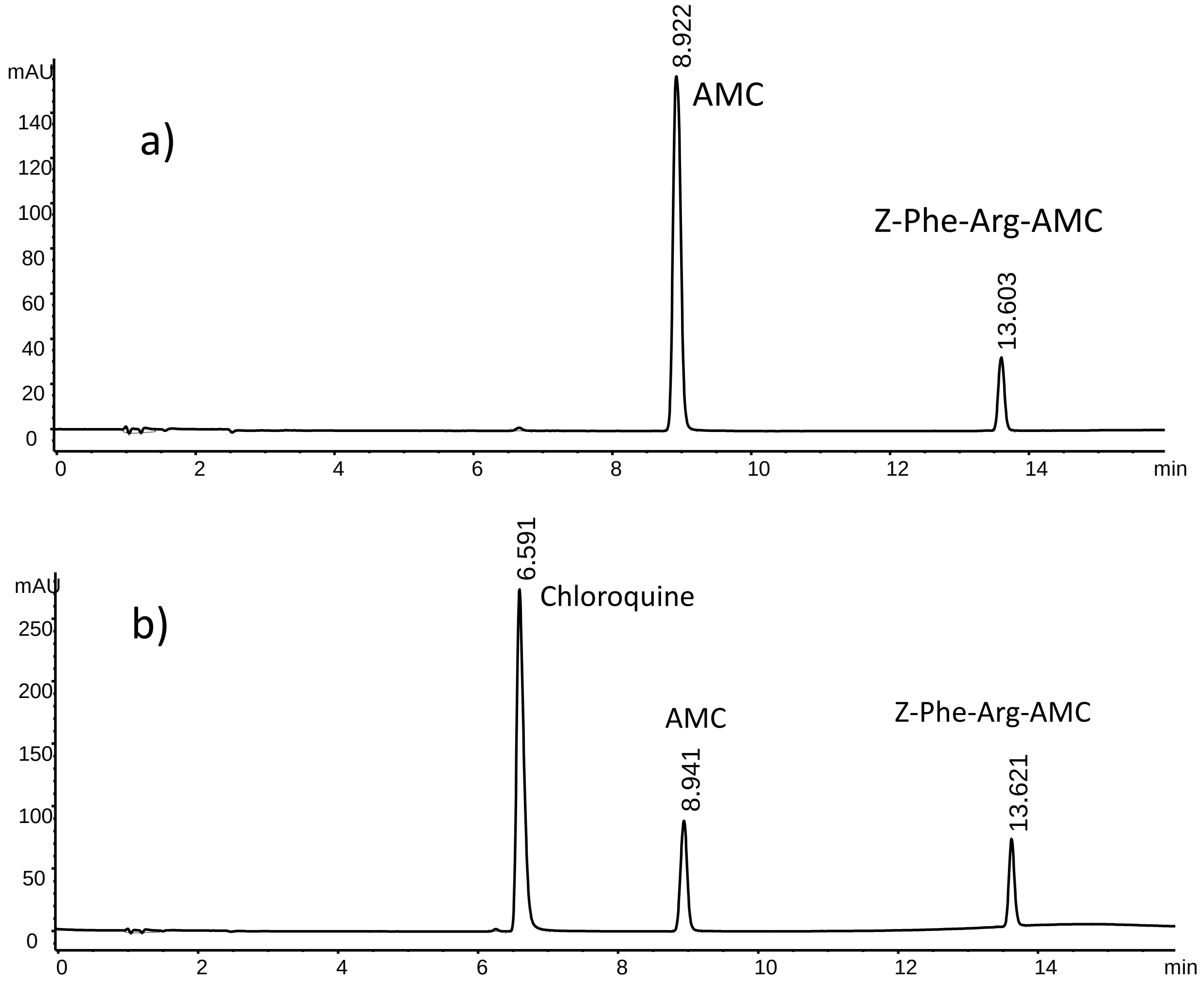

Supplementary Figure 7

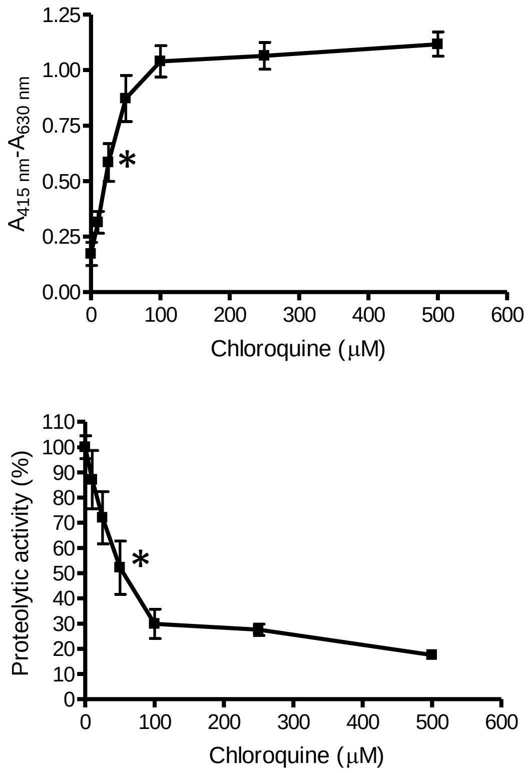

Supplementary Figure 8
